# Phytochemicals, Antioxidant Activity and Ethnobotanical Uses of *Balanites aegyptiaca* (L.) Del. Fruits from the Arid Zone of Mauritania, Northwest Africa

**DOI:** 10.3390/plants9030401

**Published:** 2020-03-24

**Authors:** Selouka Mint Abdelaziz, Fouteye Mint Mohamed Lemine, Hasni Ould Tfeil, Abdelkarim Filali-Maltouf, Ali Ould Mohamed Salem Boukhary

**Affiliations:** 1 Université de Nouakchott Al Aasriya, Faculté des Sciences et Techniques, Unité de recherche génomes et milieux, nouveau campus universitaire, Nouakchott, P.O. Box 880, Mauritanie; selouka@ymail.com (S.M.A.); fouteye@yahoo.fr (F.M.M.L.); 2 Laboratory of Microbiology and Molecular Biology, Faculty of Sciences, Mohammed Vth University, Rabat 10100, Morocco; filalimaltouf@gmail.com; 3 Laboratoire de chimie, Office national d’inspection sanitaire des produits alimentaires (ONISPA), Nouakchott P.O. Box 137, Mauritanie; hasni2002002@yahoo.fr

**Keywords:** *Balanites aegyptiaca*, the desert date, phytochemicals, antioxidant activity, folk medicine, aridity, Mauritania, Sahel, Sahara

## Abstract

Phytochemicals and antioxidant activity of fruits of 30 *B. aegyptiaca* trees naturally growing in the hyper-arid and arid zones in Mauritania were evaluated by following standard procedures. Ethnobotanical uses of fruit pulps and kernel were assessed using a structured questionnaire. *Balanites aegyptiaca* fruit pulp is a good source of sugars (33 g/100 g dry matter (DM)), polyphenols (264 mg GAE/100 g DM) and flavonoids (34.2 mg/100 g DM) with an average antioxidant activity of 519 µmol TEAC/100 g DM. The fruit kernel is rich in lipids (46.2 g/100 g DM) and proteins (29.5 g/100 g DM). Fruits from the hyper-arid zone exhibited high level of polyphenols, antioxidant activity and soluble tannins. Almost all of the informants (97.14%) reported the use of fruit pulp in folk medicine to treat diabetes, while 72.86% reported using the fruit pulp to treat hypertension. Kernel oil is mainly employed as ointments in the treatment of paronychia (57.14%) and dermal infections (35.71%). The predominant methods for preparing/administering fruit pulp/Kernel were maceration (58.8%), sucking fruit pulp (25.7%) and decoction (24.2%). *Balanites aegyptiaca* fruit contain both nutritional and health-promoting phytochemicals that could be of interest in the development of strategies for sustainable use of this neglected indigenous fruit tree.

## 1. Introduction

*Balanites aegyptiaca* (L.) Del. is an evergreen dicotyledonous multibranched Savannah tree species native to arid and semi-arid areas of Africa, the Arabian Peninsula and South Asia [1]. The question of whether the genus *Balanites* is a member of the family *Zygophyllaceae* or the family *Balanitaceae* is still a matter of continuing controversy among scientists [2,3,4,5].

*Balanites aegyptiaca*, known in Mauritania as Teichot, tolerates drought and salinity, and thrives on a wide variety of soil types [6,7,8]. The tree is highly appreciated throughout Sahelian and Saharan regions for its woods (suitable for cooking), edible fruits (sweety mesocarp and nutritive kernel oil), animal feed value, shade and shelter and numerous ethnomedicinal uses [7,9,10,11,12].

The fruit commonly known as desert date in English has different names according to the ethnic groups living in Mauritania. Thus, it is called *Toogga* in Hassaniya (the Arabic dialect spoken by the Moorish ethnic group), and *Séxéné, Mortodé* and *Sump* in Soninke, Pular and the Wolof Black African languages, respectively. It is a plum-sized and faintly five-grooved drupe consisting of epicarp (5%–9%), mesocarp (28%–33%), endocarp (49%–54%) and kernel (8%–12%) [13]. Immature fruits are green and pubescent, turning yellow to brownish and glabrous after ripening with a sweet-bitter taste. The fleshy pulp of the desert date fruit contains a high amount of sugars (35%–42%) of which 81.3%–91.1% is present as reducing sugars [14,15]. The fruit kernel contains a considerable amount of nutrititve oil (40%–51%) made up of 11 different fatty acids comprising both saturated (mainly palmitic and stearic) and unsaturated (predominantly oleic and linoleic) [13,16,17,18]. The fruit kernel is also rich in proteins (26.1%–34.3%) [13,18] and is reported to contain a variable amount of polyphenols (47.8–117.5 mg/100 g) [8]. However, saponins, referred to as balanitinis, remain by far the most reputed chemical constituents of the *B. aegyptiaca* fruit, representing 7.2% in the pulp and 6.7% in the kernel [19]. Saponins are steroidal glycosides (steroidal sapogenins), which yield diogsenin, a precursor for the synthesis of cortisones, oral contraceptives and other steroidal drugs in the pharmaceutical industry [9,20,21,22]. Other bioactive compounds such as alkaloids, flavonoids, tannins and vitamins have been reported in the fruit as well as the leaves, branches and roots of the desert date [2,15,22].

Moreover, numerous ethnopharmacological surveys showed that all of the parts of *B. aegyptiaca* have medicinal properties; most notable is the use of the fruit pulp as an antidiabetic medicine in Egypt and Sudan [18,23], and against helminthes infection [24], while the seed extract is used against Bilharzia [25]. The root is used in the treatment of abdominal pains and asthma [24]. The bark of the plant is used as a remedy for malaria and syphilis and also as an antijundice agent [22]. In the neighboring country of Senegal, squeezed pulp is used to treat gastric ulcer, hypertension and constipation [12]. In addition to the above-mentioned ethnomedicinal uses, saponin fractions of the desert date fruits’ mesocarp were shown to possess high larvicidal activity against the *Aedes aegypti* mosquito, the major vector of dengue fever [26].

Due to its high ecological plasticity and economic interest, *B. aegyptiaca* was recently suggested among other tree species native to the saharo-sahelian ecosystem to build the so called “great green wall”, which is part of the Sahara and Sahel initiative aiming at the restoration of the forest landscapes and degraded lands [27].

Natural populations of *B. aegyptiaca* are common in the Sahelian and Saharan ecoclimatic zones of Mauritania [28]. Although the tree is considered one of the popular trees in the Mauritanian folk medicine, empirical knowledge on its health benefits is still transmitted in oral form among local communities and the phytochemicals and functional potentials of this neglected indigenous fruit tree are not yet fully explored. The purposes of this study were to evaluate the phytochemicals and the antioxidant potential of the fruit of *B. aegyptiaca* trees naturally growing in the hyper-arid and arid zones in Mauritania, and assess its ethnobotanical uses. This could help in developing new economic opportunities for rural communities.

## 2. Results and Discussion

### 2.1. Descriptive Statistics Analysis of Physicochemical Variables

Morphological and physicochemical characteristics of the fruit were measured for all the collected *B. aegyptiaca* trees and the average, minimum and maximum values, as well as the standard deviations and coefficients of variations were reported for each study site and over all the study sites. Results are summarized in Table 1 and Table 2. The pH of the fruit pulp exhibited the lowest coefficient of variation (3.2%) and titratable acidity the highest (58.8%) (Table 1). Over the 13 morphological and physicochemical variables studied, lipids content, pH and proteins content exhibited coefficients of variation lower than 10%, fruit length and fruit width showed coefficients of variation between 10% and 20% and the remaining variables (fruit weight, moisture content, total sugars, titratable acidity, soluble tannins, polyphenols, flavonoids and antioxidant activity) exhibited variation values higher than 20% (Table 1). The values of coefficient of variation in fruit species are generally considered low below 10%, medium ranging from 10% to 20% and high above 20% [29].

Means comparison of the fruit morphological and physicochemical properties of *B. aegyptiaca* trees from the five sites are presented in Table 2.

The significantly highest average fruit weight was observed in trees from the arid site of Aghchorguit with 5.8 ± 0.9 g. Fruit weight from the other sites showed comparable values. Trees from the hyper-arid site of Yaghref and the arid site of Aleg showed the lowest values of fruit length (19.5 ± 1.1 mm) and diameter (15.8 ± 1.9 mm), respectively, and those from Aleg (25.7 mm) and Aghchorguit (20.5 mm) the highest. Overall, the average fruit weight (4.56 g), length (23.7 mm) and diameter (19.0 mm) found in this study are comparable to those found in countries where *B. aegyptiaca* naturally occurs such as Senegal [30] and Niger [31]. For instance, *B. aegyptiaca* fruits from Senegal possessed weight, length and diameter averages of 4.03 g, 24.4 mm and 18 mm, respectively [30]. However, values reported from Sudan were higher regarding fruit weight (7.1 g), length (27.7 mm) and diameter (21.6 mm), than those reported in the present study [8]. Variations in fruit morphology among populations of the same species have been reported in other tree species such as *Tamarindus indica* [32] and *Acacia Tortilis* [33]. The observed variations could be the results of adaptive evolution in response to different factors such as environmental and climatic conditions. 

The average sugar content was significantly higher in the fruit pulps from the hyper-arid site of Yaghref (45.8±7.1 g/100 g dry matter (DM)) compared to the other sites (Table 2). Sagna et al. [15] found a comparable value (42.60 ± 0.63 g/100 g DM) in the dry pulp of *B. aegyptiaca* fruit from Senegal. However, Soloviev et al. [30] reported lower sugar contents (13 g/100 g DM). The difference in the method of analysis used, the age of the mature fruit and the agro-ecological origins of the desert date accessions studied could explain these variations. The comparison with other saharo-sahelian indigenous fruit trees showed that the sugars content of *B. aegyptiaca* fruit pulp was higher than those of wild Jujube (*Ziziphus mauritiana*) (20.5g/100 g DM) and the Baobab (*Adansonia digitata*) (7.2 to 11.8 g/100 g DM) [30,34]. The moisture contents of fruit pulp from the arid sites of Aleg (28.1%) and Aghchorguit (24.6%) were higher than those of the hyper-arid site of Yaghref (20.3%) and Chami (21.1%). These results are close to those reported by Soloviev et al. [30] in *B. aegyptiaca* fruit from Senegal (14.6 to 24.1%), but slightly higher than that found by Sagna et al. [15], who reported an average of 16% humidity. There is a close relationship between the sugar and moisture contents in some fruits. For instance, the ratio of total sugar to moisture content expressed as a quality index (QI) has been used to classify the date palm (*Phoenix dactylifera*) fruits into soft (QI < 2), semi-dry (2 < QI < 4) and dry (QI > 4) [35].

Accordingly, *B. aegyptiaca* fruits analyzed in the present study showed a mixture of soft and semi-dry fruits regarding their QI (Aleg = 1.2; Aghchorguit = 1; Boutilimit = 1.8; Yaghref = 2.2; Chami = 1.4). A quality index of 2.6 was reported in some fruits of *B. aegyptiaca* from Senegal [15]. 

Titratable acidity expressed as equivalent citric acid was significantly higher (0.3 ± 0.1 g/100 g DM) in the trees from Aghchorguit (arid zone) and Yaghref (hyper-arid zone). This value is in accordance with the quasi-absence of acidity from the flavor of the study fruit pulp. Pulp juice with the lowest pH values (4.7 ± 0.1) was that of the Aleg trees. Fruits from Aghchorguit (arid zone) and Yaghred and Chami (hyper-arid zone) shared the highest pH values of pulp juice (4.9 ± 0.1). 

Significant differences were observed between the accessions for the total kernel oil (Table 2). It ranged between 43.3 g/100 g DM in the Aleg fruit accessions and 48.9 g/100 g DM in the Yaghref accessions. Fruit accessions from Aghchorguit contained a significantly high level of proteins (32.5 ± 0.4 g/100 g DM) compared to the other accessions (28.1–29.2 g/100 g DM). These results are in close accordance with those reported by Ahmed et al. [8] in eight Sudanese desert date accessions (47.3% for lipids and 30% for proteins) and by Mohamed et al. [13], which reported values of 49% and 32.4% for lipids and proteins content, respectively, from one Sudanese fruit accession. The present study showed that the fruit kernel of *B. aegyptiaca* contains more lipids than that of some oleaginous trees of agro-industrial importance like the Argan tree (*Argania spinosa*) that possessed 36% lipids [36]. This finding demonstrates the great socio-economic potential of this species beside its ecological importance in combating desertification in the Sahel and Sahara.

### 2.2. Bioactive Compounds and Antioxidant Activity of Fruit Pulp

Bioactive compounds and antioxidant activities of *B. aegyptiaca* fruits varied significantly between the study sites (Table 3). Fruit pulp from the hyper-arid sites of Yaghref and Chami showed the highest phenolic contents (396 ± 4.8 and 268 ± 1.9 mg GAE/100 g DM, respectively) and antioxidant activities (750 ± 5.5 and 730 ± 10.5 µmol TEAC/100 g DM, respectively). They also showed the highest values of the soluble tannins (714 ± 17.3 and 756 ± 8.4 mg TAE/100 g DM, respectively). However, total flavonoids were significantly higher in the fruit of the arid site of Boutilimit (47.4 ± 6.4 mg QE/100 g DM) than the other sites. Literature data showed a wide range of bioactive compounds and antioxidant potential of *B. aegyptiaca* fruit pulp of various origins [37,38,39,40,41]. Polyphenol and flavonoid contents found in the present study were lower than those reported by Abdallah et al. [37] in methanolic extracts of *B. aegyptiaca* fruit from Sudan (212 mg GAE/g DM and 11.5 mg QE/g DM, respectively). These authors also showed a dose-dependent scavenging activity of pulp extract against the 2,2-diphenyl-1-pyrrylhydrazyl (DPPH)-radical with a half maximal inhibitory concentration (IC50) of 3µg/mL. 

Correlation between bioactive compounds and antioxidant activity was not examined in the present study. However, literature data showed that the antioxidant potential of many medicinal plants was generally correlated to the presence of polyphenols and/or flavonoids [40]. 

### 2.3. Principal Component Analysis (PCA)

**Figure 1:**
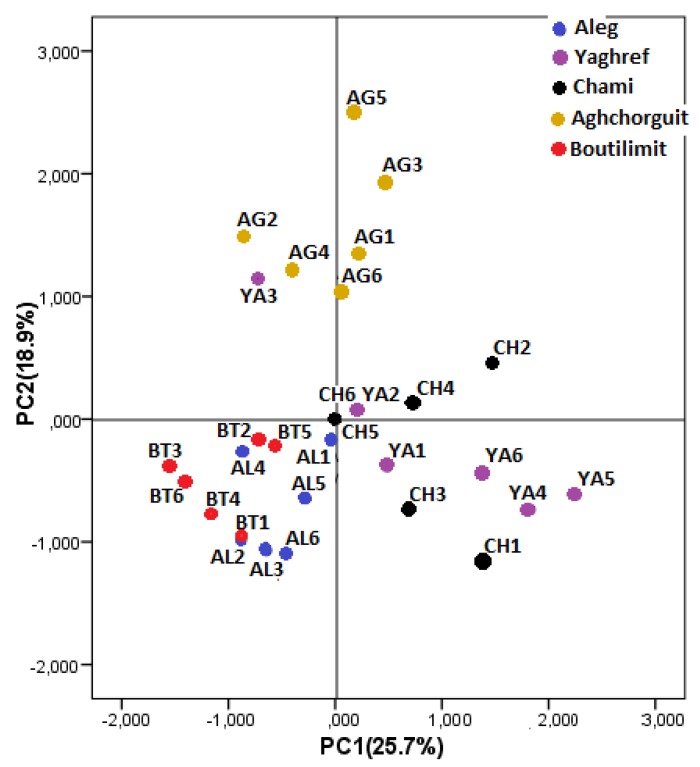
Principal component analysis plots (axis 1–2) of 30 *Balanites aegyptiaca* trees from 5 locations in Mauritania based on fruit morphological and phytochemical components.

The PCA was applied to all physical and chemical variables to determine variables that differentiate the desert date fruit from different sites and to identify any group patterns. Three principal components (PC1, PC2 and PC3) explain 59% of the total variance (25.7%, 18.9% and 14.4% for PC1, PC2 and PC3, respectively) (Table S1). Total polyphenols (0.85), antioxidant activity (0.92) and soluble tannins (0.83) were high positively correlated with the PC1, while fruit length (−0.5) was negatively correlated with the PC1. The factors that contributed most positively to the PC2 were proteins content of the fruit kernel (0.66), fruit width (0.62) and fruit weight (0.82), and that contributing most negatively was total sugars (−0.68). The PC3 was positively correlated to the lipid contents (0.84) and negatively correlated to the moisture content (−0.69). The PC1 versus PC2 scatter plot (Figure 1) clearly distinguished two main clusters: the first one grouped *B. aegyptiaca* trees from the hyper-arid sites of Chami and Yaghref characterized by high polyphenols contents, important antioxidant activity and high soluble tannins compared to *B. aegyptiaca* trees from the arid sites. The second cluster consisting of trees from the arid site of Aghchorguit, which are characterized by high fruit weight, important proteins content of the fruit kernel and large fruit. Based on the PCA analysis, fruits from the hyper-arid zone (Yaghref and Chami) appeared the most interesting regarding the bioactive compounds and antioxidant activity. 

### 2.4. Ethnobotanical Uses of Balanites Aegyptiaca Fruits

A total of 70 informants including seven traditional healers were interviewed: 43 (61.4%) men and 27 (38.6%) women (P = 0.064), ranging in age between 30 and 70 years (mean age = 50; median age = 53) (Table 4). In general, interest and therefore knowledge in medicinal plants generally increases with age as shown in a similar study [42]. Respondents originated from seven out of the thirteen provinces of the country, including Nouakchott, and belong to the main ethnolinguistic groups of Mauritania: Moorish of Arab descent (87.1%) and black Africans (12.9%). The educational background of the respondents consisted of 27 (38.6%) with tertiary educational level, while 16 (22.8%) and 13 (18.6%) reached secondary and primary educational levels, respectively, and 14 (20%) had no formal educational level. Of the 70 respondents, 52 (74.3%) actively use plant-based medicine as a first line to treat their ailments against 18 (25.7%) that prefer conventional medicine as a first recourse.

All informants recognized the fruit of *B. aegyptiaca* and gave its correct name in their native language (Table 5). However, only 20% (14/70) indicated exactly that the best period to collect mature fruits from the wild is in the winter (December–February). The most commonly used plant part of the desert date fruit is the pulp, either as food or as medicine (Table 5). Traditional kitchen is the main reported culinary use (74.3%) of the desert date fruit. Using the pulp in pastry was also reported in 8.6% of the informants. Most of the participants reported using fruit pulp as medicine to treat diabetes (97.1%), hypertension (72.8%), constipation (40%) and cough (35.7%) while they employed the fruit kernel to treat paronychia (57.1%), various dermal diseases (35.7%) and asthma (25.71%). The preparation/administration methods included maceration (58.8%), decoction (28.5%), sucking fruit pulp (25.7%) and ointment (12.8%). The majority of respondents reported using three fruit pulps once a day (53.4%) during the period of treatment. 

The present ethnobotanical survey revealed that desert date fruit provides remedies for 8 human ailments according to the interviewed individuals, including diabetes, hypertension, constipation and cough. The study also reported the use of the fruit pulp for culinary purposes. Similar traditional medicinal and culinary uses of *B. aegyptiaca* fruit were also reported elsewhere. For instance, in Senegal, fruit pulp is used to treat human illness such as hypertension [12], in Egypt and Sudan to treat diabetes [43] and in Burkina Faso, Algeria and Senegal to treat constipation [10]. In Senegal, culinary uses of fruit pulp and kernel were reported [12]. The similarity in the medicinal and culinary uses of *B. aegyptiaca* fruit pulp between populations living in the Sahel and Sahara including the Mauritanians is a demonstration of the nutritional value, as well as the medicinal and pharmacological potentialities of the plant.

Biological evidence from in vivo and in vitro studies supported the potential medicinal virtues of the desert date fruits [37,40,44,45]. For instance, aqueous extract of *B. aegypticea* fruit demonstrated hypoglycemic properties in diabetic experimental rats [45]. It was also reported that whole and extracted pulp of *B. aegyptiaca* fruits exhibited a hypocholesterolemic effect when tested on adult albino rats [44]. Moreover, methanol extract of the pulp was also found to exhibit anti-dermatophytic activity on *Microsporum gypseum* and *Trichophyton rubrum* [40]. Fixed fruit oils exhibited anticancer activity against lung, liver and brain human carcinoma cell lines, possessed anthelmintic activity against hepatic worms (*Schistosoma mansoni* and *Fasciola gigantica*) and showed antimicrobial activity against selected strains of Gram-positive and Gram-negative bacteria [17]. Ethanolic extract of the roots of *B. aegyptiaca* possess a bioactive compound that exerts sedative and anxiolytic properties in mice [46]. It is worth noting that most of these studies reported the active compounds to be saponins.

## 3. Materials and Methods

### 3.1. Study Sites

**Figure 2:**
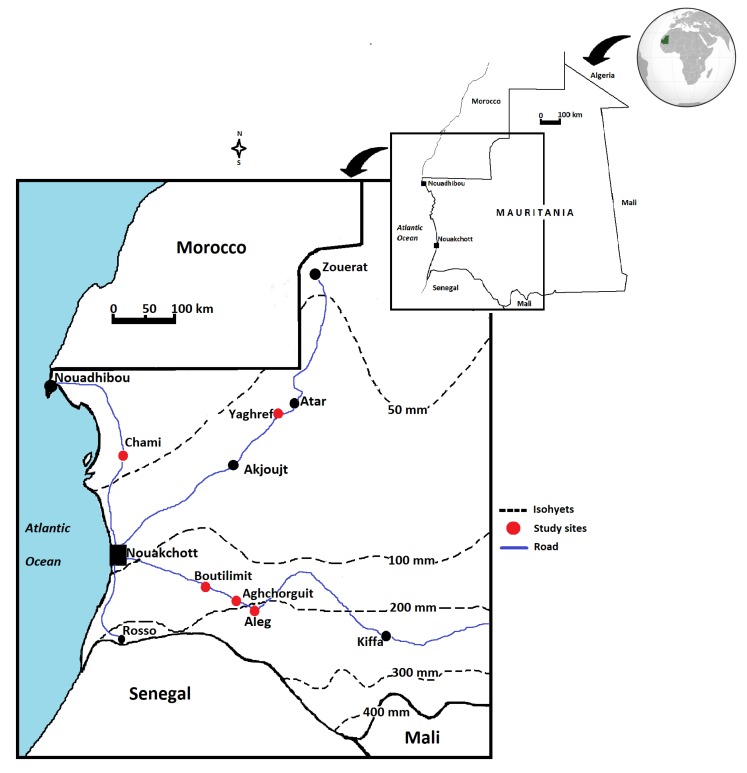
Distribution map of the five locations of *Balanites aegyptiaca* trees in the central arid (Aleg, Aghchorguit and Boutilimit) and northern hyper-arid (Yaghref and Chami) zones in Mauritania, Northwest Africa. Professor Ali Ould Mohamed Salem Boukhary created Figure 2 by using the Microsoft Paint application.

Five study sites (Aleg, Aghchorguit, Boutilimit, Yaghef and Chami) located in the central arid and northern hyper-arid zones in Mauritania where prospected (Figure 2). Annual rainfall in the study sites follows a south–north gradient with an average of 200 mm over the arid sites of Aleg and Aghchroguit and 50 mm or less along the hyper-arid sites of Chami and Yaghref. *Balanites aegyptiaca* trees abundance across the study zone follows the rainfall gradient with Aleg showing the highest density and Chami and Yaghref the lowest. These later represent the northernmost limit of distribution area of the desert date tree in Mauritania. In each study site, sampling was performed within a distance of hundreds of meters from both sides of the national roads connecting Nouakchott, the capital city, and Aleg to the southeast, Nouakchott and Nouadhibou to the north and, Nouakchott and Atar to the northeast (Figure 2). 

### 3.2. Sampling and Measurement of Fruit Morphological Variables

Fruits were collected during the harvest seasons of 2016 and 2017, from 30 *B. aegyptiaca* trees (six trees from each site) naturally growing in the study sites. After spreading a plastic sheet around the trunk of the tree, fruits from each tree were collected by shaking the upper tree branches using a long stick and 30 among the fully mature dropped fruits were randomly selected and mixed as a single lot. They were then rapidly transported in a cooler to the laboratory where fruit morphological variables were immediately measured. Fruit collections were then stored at −20 °C for further phytochemical characterization.

Ten fruits from each lot were used to describe fruits. Fruit weight was measured with an accuracy of 0.01 g using an analytical balance (Ohaus, Heuwinkelstrasse, Switzerland). Fruit length and diameter were measured to the nearest 0.1 mm using a digital calliper.

### 3.3. Phytochemical Analysis

A total of 10 physicochemical variables related to fruit pulp (mesocarp) (eight parameters) and fruit kernel (two variables) were measured.

Moisture content was determined by drying 1 g of fruit pulp in an oven at 65 °C until the weight stabilized, and the moisture percentage was calculated according to the following formula:

Moisture (%) = [(fresh weight − dry weight)/fresh weight] × 100.

Titratable acidity and pH were estimated by homogenizing 10 g of fruit pulp in 100 mL distilled water. The pH of 25 mL of the resultant pulp juice was estimated using a digital pH meter (Hanna instruments HI84435-01 Mini Titrator and pH Meter) previously calibrated with buffered solutions (pH 4.0 and 7.0). Titratable acidity was estimated on 50 mL of the same juice pulp by titration to pH 8.0 using a 0.1 N NaOH solution and a phenolphthalein indicator [47]. The results were expressed as grams citric acid equivalents per 100 g dry matter.

Sugars were extracted from 1 g of dried fruit pulp using 80% ethanol aqueous solution and quantified according to the spectrophotometric phenol-sulfuric acid method developed by Dubois et al. [48]. A calibration curve was prepared using glucose solution as a standard. Absorbance was measured at 490 nm. Sugars content was expressed in grams of glucose equivalents per 100 g dry matter.

To quantify kernel oil and proteins, fruit external tissues (epicarp and mesocarp) were removed manually using a thin sharp knife and the kernel was separated from the enclosing woody part after gentle hand hammering. Total fats were extracted according to the Soxhlet method using hexane as the extraction solvent [47]. One gram of homogenized fruit kernel, 10 g sea sand and 200 mL hexane were extracted by Soxhlet for 6 h. Then, the solvent was evaporated at 35 °C using a rotary evaporator. Finally, the lipid content of the samples was determined gravimetrically.

Proteins content in the fruit kernel was determined according to the Kjeldahl method as described in the Association of Official Agricultural Chemists (AOAC) International [49]. Briefly, 1 g of fruit kernel was digested with 15 mL concentrated sulfuric acid containing two copper catalyst tablets in a heat block (Kjeltec system 2020 digester, Tecator Inc., Herndon, VA, USA) at 420 °C for 2 h. After cooling, distilled water was added to the hydrolysates before neutralization and titration. The amount of total nitrogen in the pulp was multiplied with the traditional conversion factor of 6.25 to convert the measured nitrogen concentration to a protein concentration. 

Polyphenols and flavonoids were extracted from 1 g of dried fruit pulp using 80% methanol aqueous solution. Polyphenols were quantified using Folin–Ciocalteu’s reagent according to the colorimetric method of Singelton and Rossi [50] as modified by Kim et al. [51]. A standard curve was prepared with gallic acid. The absorbance was read at 750 nm and results were expressed as mg of gallic acid equivalents (GAE) per 100 g dry matter. Flavonoids were quantified using Aluminium chloride colorimetric assays according to the protocols described by Zhishen et al. [52] and Kim et al. [51]. The absorbance of the tested samples and standard quercetin solutions were measured at 430 nm. Results were expressed as mg quercetin equivalents (QE) per 100 g dry matter. 

Soluble tannins were extracted from 1 g of dried fruit pulp using 70% acetone aqueous solution. Soluble tannins were estimated using the Folin–Denis colorimetric method as described by Taira [53]. This method is based on the reduction of phosphomolybdic-phosphotungstic acids by tannins to molybdenum blue in alkaline solution. A calibration curve was constructed using tannic acid in acetone aqueous solution. The absorbance was measured at 760 nm. Tannins content was expressed as mg tannic acid equivalents (TAE) per 100 g dry matter.

### 3.4. Antioxidant Activity of Fruit Pulp

Antioxidant activity of fruit pulp methanolic extracts was estimated using the 2,2-diphenyl-1-pyrrylhydrazyl (DPPH) free radical assay as described by Brand-Williams et al. [54] and Mint Mohamed Lemine et al. [55]. The Absorbance was measured at 517 nm. The antioxidant activity was given in µmol of trolox equivalents antioxidant capacity (TEAC) per 100 g of dry matter.

### 3.5. Ethnobotanical Uses of the Desert Date Fruit

The survey took place during June–July 2019 in Nouakchott, the capital city of Mauritania. Ethnobotanical uses of the desert date fruit were assessed using a structured questionnaire with close-ended multiple-choice questions translated into the participants’ native languages (Hassaniya, Pular, Soninke and Wolof). The survey was conducted face-to-face in order to maximize response rates [56]. Before obtaining the verbal consent, the aims and objectives of the survey were explained to each prospective respondent. The questions included the local name of the fruit, the harvest period, the culinary and medicinal uses, the mode of preparation/administration and the frequency of use. The questionnaire also comprised socio-demographic data on each informant (age, gender, ethnicity and education). Interviews were carried out at homes, in the street or in different local markets in Nouakchott. Seven traditional healers having practical knowledge of medicinal plants were also included in the survey. 

### 3.6. Data Analysis

All measurements were conducted on three replicate samples. Means ± standard deviation (SD), minimum, maximum values and the coefficient of variation for each physicochemical variable were calculated. Means of the measured variables were compared among the study sites using the Student–Newman–Keuls test [57]. Principal components analysis (PCA) was performed on the collected data to assess the relationships between desert date trees from the different study sites. Information obtained through the ethnobotanical survey was analyzed and expressed as percentages. All statistical analyses were carried out using SPSS software (Version 12) or MS Excel (2007). The significance level was accepted at *p* < 0.05. 

## 4. Conclusions

This is the first study on the phytochemical properties and ethnobotanical uses of the desert date fruit at the northern edge of its distribution area in Mauritania. Results show important diversity in their fruit phytochemicals and antioxidant activity, and highlight a body of indigenous ethnobotanical and ethnomedicinal uses that constitute a basis for the development of strategies of conservation and sustainable use of these underutilized genetic resources. 

## Figures and Tables

**Table 1:** Descriptive statistics of 13 physicochemical parameters measured in fruits of 30 *Balanites aegyptiaca* trees from five locations in Mauritania, Northwest Africa.

Parameter (Unit)	Min	Max	Mean	SD	CV (%)
**Fruit morphology**					
Fruit length (mm)	18.5	29.3	23.7	3.1	13
Fruit diameter (mm)	13.5	23.0	19.0	2.2	11.5
Fruit weight (g)	3.5	6.8	4.5	0.9	21
**Pulp characteristics**					
Moisture content (%)	5.41	30.1	21.9	12.2	56
Total sugars (g/100 g DM)	17.5	54.0	33	9.1	27.5
pH	4.43	5.1	4.9	0.16	3.2
Titratable acidity (g/100 g DM)	0.01	0.35	0.17	0.1	58.8
Soluble tanins (mg TAE/100 g) DM	280	971	559.6	191.6	34.2
Total polyphenols (mg GAE/100 g DM)	173	429	265	75.3	28.4
Flavonoids (mg QE/100 g DM)	14.7	63.2	34.2	15.9	46.4
Antioxidant activity (µmol TEAC/100 g DM)	220	800	519	176	34
**Kernel characteristics**					
Lipid content (g/100 g DM)	42.6	49.7	46.2	2.1	4.5
Protein content (g/100 g DM)	27.5	32.7	29.5	1.8	6.1

Abbreviations: DM: dry matter; CV: coefficient of variation; SD: standard deviation; TAE: tannic acid equivalent; GAE: gallic acid equivalent; QE: quercetin equivalent; TEAC: trolox equivalent antioxidant capacity.

**Table 2:** Means ± standard deviation of some physicochemical variables measured in fruits of 30 *B. aegyptiaca* trees from five locations in Mauritania, Northwest Africa.

Study Site	Fruit Weight (g)	Fruit Length (mm)	Fruit Width (mm)	Moisture Content (%)	Total Sugars (g/100 g DM)	Protein Content * (g/100 g DM)	Lipid Content * (g/100 g DM)	pH	Titratable Acidity (g/100 g DM)
Aleg	4.1 ± 0.5 ^a^	25.7 ± 3.4 ^a^	15.8 ± 1.9 ^a^	28.1 ± 9.0 ^a^	35.4 ± 5.3 ^a^	28.3 ± 1.1 ^a^	43.3 ± 1.0 ^a^	4.7 ± 0.1 ^a^	0.2 ± 0.05 ^a^
Aghchorguit	5.8 ± 0.9 ^b^	25.2 ± 1.4 ^a^	20.5 ± 2.1 ^b^	24.6 ± 2.6 ^a^	23.6 ± 1.5 ^a^	32.5 ± 0.4 ^b^	45.1 ± 1.3 ^b^	4.9 ± 0.1 ^b^	0.3 ± 0.1 ^b^
Boutilimit	4.5 ± 1.0 ^a^	23.5 ± 3.1 ^a^	19.4 ± 1.2 ^b^	18.3 ± 4.0 ^b^	32.5 ± 5.3 ^b^	29.2 ± 0.2 ^a^	46.4 ± 0.2 ^c^	4.8 ± 0.2 ^a,b^	0.1 ± 0.02 ^c^
Yaghref	4.0 ± 0.2 ^a^	19.5 ± 1.1 ^b^	19.7 ± 0.8 ^b^	20.3 ± 5.7 ^a,b^	45.8 ± 7.1 ^c^	28.1 ± 0.1 ^a^	48.9 ± 1.1 ^d^	4.9 ± 0.1 ^b^	0.3 ± 0.1^b^
Chami	4.4 ± 0.4 ^a^	23.9 ± 0.7 ^a^	19.4 ± 2.8 ^b^	21.1 ± 3.0 ^a,b^	29.5 ± 8.1 ^a^	ND	ND	4.9 ± 0.1 ^b^	0.1 ± 0.02 ^c^

* Proteins and lipid content were measured in the fruit kernel. ND: not determined. Means in the same column sharing the same letter(s) are not significantly different at (*p* < 0.05) according to the Student–Newman–Keuls test.

**Table 3:** Means ± standard deviation of bioactive compounds and antioxidant activity of *B. aegyptiaca* fruit pulp from 5 locations in Mauritania, Northwest Africa.

Study Site	Total Polyphenols (mg GAE/100 g DM)	Total Flavonoids (mg QE/100 g DM)	Soluble Tannins (mg TAE/100 g DM)	Antioxidant Activity (µmol TEAC/100 g DM)
Aleg	245 ± 8.1 ^a^	28.8 ± 5.4 ^a^	471 ± 8.8 ^a^	440 ± 7.5 ^a^
Aghchorguit	236 ± 5.7 ^b^	32.5 ± 1.2 ^a,b^	479 ± 12 ^a^	490 ± 13.8 ^b^
Boutilimit	222.5 ± 2.5 ^c^	47.4 ± 6.4 ^d^	436 ± 9.9 ^b^	340 ± 10.2 ^c^
Yaghref	396 ± 4.8 ^d^	34.3 ± 1.8 ^b^	714 ± 17.3 ^c^	750 ± 5.5 ^d^
Chami	268 ± 1.9 ^e^	19.7 ± 2.2 ^c^	756 ± 8.4 ^d^	730 ± 10.5 ^e^

Means in the same column sharing the same letter(s) are not significantly different at *p* < 0.05 according to Student–Newman–Keuls test; GAE: gallic acid equivalent; QE: quercetin equivalent; TAE: tannic acid equivalent; TEAC, trolox equivalent antioxidant capacity; DM: dry matter.

**Table 4:** Sociodemographic characteristics of the 70 participants in the ethnobotanical survey from Nouakchott, Mauritania, Northwest Africa.

Characteristics	No.	%
Gender (n = 70)		
Male	43	61.4
Female	27	38.6
Age groups (year) (n = 70)		
30–40	19	28.6
41–50	21	30
> 50	30	41.4
Ethnicity (n = 70)		
Moors	61	87.1
Black Africans	9	12.9
Educational level (n = 70)		
No formal education	14	20
Primary education	13	18.6
Secondary education	16	22.8
Tertiary education	27	38.6

**Table 5:** Ethnobotanical and medicinal uses of the fruit of *B. eagyptiaca* reported by 70 informants from Nouakchott, Mauritania, Northwest Africa.

Characteristics		Frequency (%) *
Local name of the fruit	Desert date **	70 (100)
	False answer	0
	I don’t know	0
Period of harvest	Winter (December–February)	14 (20)
	Spring (March–May)	43 (61.4)
	Summer (June–August)	12 (17.1)
	Autumn (September–November)	1 (1.4)
Taste (pulp)	Sweet-bitter	62 (88.6)
	Bitter	7 (10)
	Sweet	1 (1.43)
Culinary use	Traditional kitchen	52 (74.3)
	Pastry	6 (8.6)
	Juice	2 (2.8)
	Pastry and traditional kitchen	10 (14.3)
Medicinal uses of fruit pulp	Diabetes	68 (97.1)
	Hypertension	51 (72.8)
	Cardiovascular illnesses	1 (1.4)
	Cough	25 (35.7)
	Constipation	28 (40)
Medicinal uses of Kernel oil	Dermal infections	25 (35.7)
	Asthma	18 (25.7)
	Paronychia	40 (57.1)
Traditional method of preparation/administration	Maceration	41 (58.8)
	Sucking (fruit pulp)	18 (25.7)
	Decoction	20 (28.5)
	As ointment (kernel oil)	9 (12.8)
Frequency of use ***	Three fruit pulps once a day	37 (53.4)
	Four to seven fruits pulp a day	33 (46.5)

* Percentages who do not add up to 100 are from multiple response questions; ** the name of the fruit was given in the native language of the respondent (see main text for the correspondence); *** during the period of treatment.

## Supplementary Materials

The following are available online at https://www.mdpi.com/2223-7747/9/3/401/s1; Table S1: Eigen values and proportion of the variance explained for the three principal components of the 30 B. aegyptiaca trees based on fruit morphological and phytochemical components.
